# Unmasking digital deceptions: An integrative review of deepfake detection, multimedia forensics, and cybersecurity challenges

**DOI:** 10.1016/j.mex.2025.103632

**Published:** 2025-09-18

**Authors:** Sonam Singh, Amol Dhumane

**Affiliations:** a Dr. D.Y Patil Institute of Technology, Pimpri, Pune, India; b Symbiosis Institute of Technology, Pune, India

**Keywords:** Deepfake detection, Generative adversarial networks (GANs), Synthetic media, biometric spoofing, Cyber security threats, Multimedia forensics, AI policy frameworks, Explainable AI, Federated learning, Digital deception, Face synthesis, Speech cloning, Identity theft, Cross-dataset evaluation, Ethical AI

## Abstract

Deepfakes, which are driven by developments in generative AI, seriously jeopardize public trust, cybersecurity, and the veracity of information. This study offers a comprehensive analysis of the most recent methods for creating and detecting deepfakes in image, video, and audio modalities. With a focus on their advantages and disadvantages in cross-dataset and real-world scenarios, we compile the latest developments in transformer-based detection models, multimodal biometric defenses, and Generative Adversarial Networks (GANs). We provide implementation-level information such as pseudocode workflows, hyperparameter settings, and preprocessing pipelines for popular detection frameworks to improve reproducibility. We also examine the implications of cybersecurity, including identity theft and biometric spoofing, as well as policy-oriented solutions that incorporate federated learning, explainable AI, and ethical protections. By enriching technical insights with interdisciplinary perspectives, this review charts a roadmap for building robust, scalable, and trustworthy deepfake detection systems.

## Highlights

• Presents an in-depth review of deepfake generation and detection, highlighting AI methods such as GANs, face synthesis, and speech cloning.
• Evaluates critically the weaknesses of biometric systems and the difficulties of cross-dataset testing for deepfake detection.
• Suggest interdisciplinary solutions—such as explainable AI, federated learning, and policy frameworks—to counteract the social and cybersecurity implications of deepfakes.

## Graphical abstract

**Figure:**
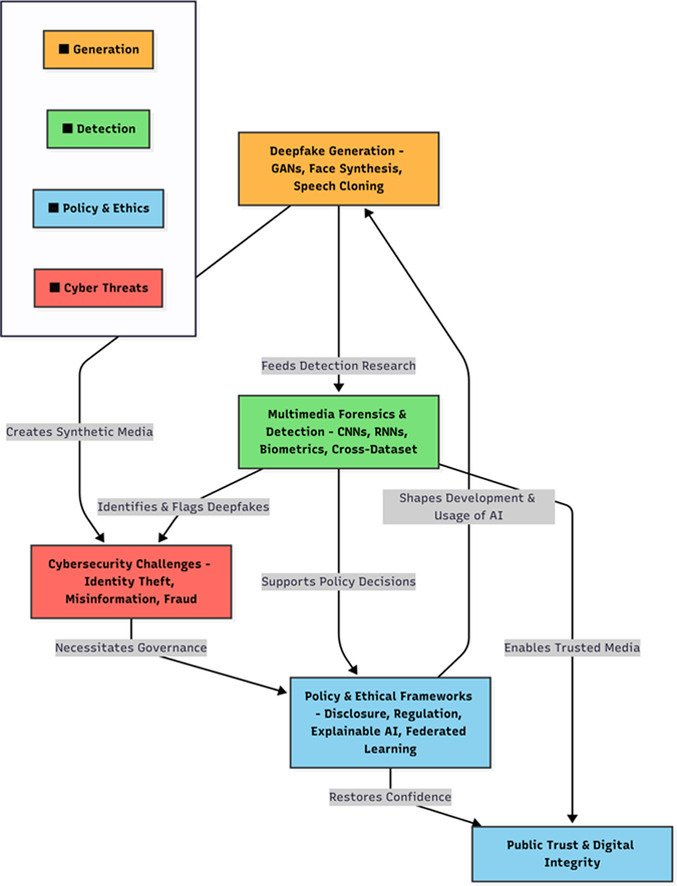

## Specifications table

**Table:** 

Subject area	Computer Science
More specific subject area	Artificial Intelligence, Deep Learning, Multimedia Forensics, and Cybersecurity
Name of the reviewed methodology	Deepfake Generation and Detection Techniques (GANs, CNN-based Detection, Biometric Spoofing Countermeasures)
Keywords	Deepfake detection; Generative Adversarial Networks (GANs); Synthetic media, Biometric spoofing; Cyber security threats; Multimedia forensics; AI policy frameworks; Explainable AI; Federated learning; Digital deception; Face synthesis; Speech cloning; Identity theft; Cross-dataset evaluation; Ethical AI
Resource availability	FFHQ, CelebA, VoxCeleb, FaceForensics++, TIMIT, DeepfakeTIMIT, Google Speech Commands, and other multimedia forensics datasets
Review question	1. What are the current state-of-the-art methodologies for generating deepfakes across image, audio, and video domains? 2. What deep learning-based and biometric-specific detection techniques exist to counter these technologies? 3. What are the vulnerabilities and limitations of these detection methods, particularly in cross-dataset evaluations? 4. How do deepfake technologies impact cybersecurity, identity verification, and policy frameworks? 5. What are the emerging trends and future directions—such as explainable AI and federated learning—for combating deepfakes effectively?

## Background

Deepfakes create incredibly lifelike synthetic media, such as audio, images, and videos, by utilizing AI and deep learning advancements [1]. It has changed a lot of industries, including entertainment, education, and healthcare, which eventually leads to new ways of experiencing creativity. Examples range from the use of it in therapy and healing in the medical field to the digital reanimation of deceased celebrities in museums. But the same technology also increased the risks of identity theft, privacy violations, and the spread of false information [2]. The widespread availability of AI tools for producing deepfakes democratized access and raised the possibility of abuse, making their control a global issue.

### Motivation

**Fig. 1:**
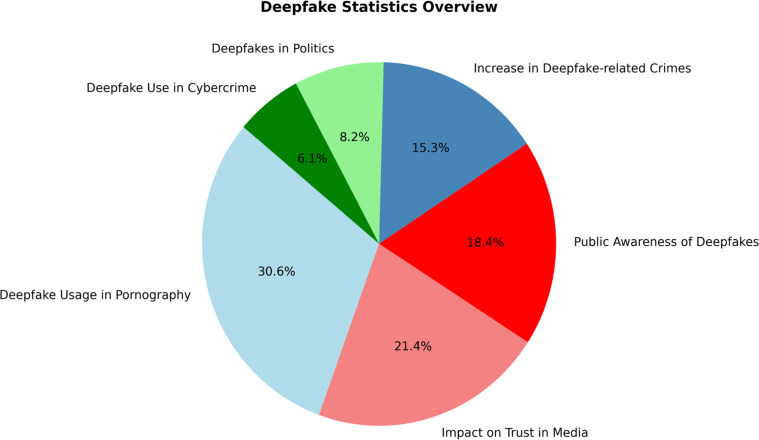
Deepfake Statistics Overview (Based on Aggregated Reports).

In the modern world, the issue of deepfakes is rapidly changing, and conversations about their use, detection, and social effects are becoming more prevalent [11]. Based on numerous global reports, including SecurityHero (2023), Keepnet Labs, the Alan Turing Institute, and Recorded Future, Fig. 1 provides a concise overview of some noteworthy statistics and trends in deepfakes. The sections discuss the social impact and practical applications of deepfake techniques in the real world [94], [95], [96], [97], [98], [99].

This escalation underscores the urgency for robust detection mechanisms and motivates the current review, which aims to consolidate recent advances and identify research gaps for future response strategies.

This review synthesizes current research efforts, with the aim of charting a roadmap for addressing these multifaceted challenges and ensuring the responsible use of deepfake technologies [13].

## Method details

### Deepfake creation techniques

Deepfakes — a term derived from the combination of "deep learning" and "fake" — refer to synthetic media generated using AI techniques. While early deepfakes often involved replacing a person in an existing image or video with someone else's likeness, recent advancements have broadened their scope significantly. Today, deepfakes can also be entirely synthetic, generated from scratch using text-to-image or text-to-video models, and are not limited to human subjects. Mostly, deepfakes technology progresses with AI growth, which recently has taken some new steps via Generative Adversarial Networks, or GANs [67]. This paper expands on the primary approaches deployed for creating deepfakes, specifically, GANs: Deep learning approaches used in the process of creating face synthesis, morphing, and speech-based deepfakes [44], [45], [46], [47], [48], and how free-use tools and datasets have contributed to the proliferation of these deepfake technologies [3].

#### Introduction to generative adversarial networks (GANs) and their contribution to deepfakes

Generative Adversarial Networks, introduced by Ian Goodfellow in 2014, are a class of machine learning frameworks that have played a very important role in creating deepfakes. A GAN is composed of two neural networks: a generator and a discriminator. These networks are engaged in a zero-sum game against each other [4]. The generator will create synthetic data that will be as close to real data as possible, and the discriminator will determine the authenticity of the generated data. Over time, the generator gets better at creating highly realistic outputs that can fool the discriminator, thereby creating convincingly realistic media [15]. In the context of deepfakes, GANs are used in the following areas:

##### Face synthesis

GANs are the most commonly used in generating hyper-realistic human faces. Models such as StyleGAN and its successors, developed by NVIDIA, can create high-resolution images of human faces that do not exist in reality [17]. StyleGAN achieves this by manipulating latent spaces to control features such as age, gender, and facial expressions, enabling precise customization of synthetic faces [5]. These techniques are foundational for creating deepfake videos where one person's face is seamlessly replaced with another's.

##### Face morphing

GANs enable face morphing by blending facial features from multiple individuals, often achieved through latent space interpolation [18]. In this process, the transition from one face to another is smoothed within the latent space, producing realistic intermediate results. Face morphing [49] has found applications in generating synthetic identities [21] and in improving the visual quality of face-swapping in videos.

##### Speech and lip-sync manipulation

Another application of GANs is in the generation of speech and synchronization of lip movements with audio [28].

For example, Wav2Lip models synthesize lip motion that seems to be precisely in sync with the input sound using GAN-based architectures [29]. This feature will be essential for creating deepfakes, in which the subject appears to say things they haven't said.

Because of their adaptability and power, GANs are now at the heart of the majority of deepfake generation pipelines [34]. However, the same advantages that make GANs more powerful also make them susceptible to abuse, which raises moral and societal issues.

#### Openly available datasets and tools that facilitate deepfakes

Open-source tools and data sets have made deepfakes more accessible to the general public. Because entry barriers are reduced, even for those who are not particularly tech-savvy, this makes it easier for people and organizations to get involved in the creation of deepfakes. Among the important resources and datasets are [66]:

##### Open-Source tools

(i) DeepFaceLab:
  DeepFaceLab is one of the most commonly used tools in making deepfakes [35]. It is friendly, user-friendly, and can perform face swaps as well as face reenactment [31]. Modularity makes it very flexible to be able to work on different workflow creations, and so it suits users of both types, whether beginners or professionals [25].
(ii) Faceswap:
  Another open-source tool that is commonly used includes Faceswap, which swaps faces of individuals in videos and pictures [32]. Developed using TensorFlow, the tool has comprehensive documentation and has a large community that supports it, making it suitable for a wide audience [33].
(iii) First OrderMotion Model:This tool specializes in creating deepfakes from one image. In this, motion patterns learned from a source video can animate images in a photorealistic fashion, and is popularly utilized for applications that include the making of talking-head videos. There are more tools available in the market, as shown in Table 1 below [4], [5], [16], [17], [18], [21], [67].

**Table 1:** Commonly used Deepfake generation models.

Model	FID(Frechet Inception Distance) Score (Lower is Better)	Inference Speed (fps)	Strengths	Weaknesses	Purpose
Vanilla GAN	65.0	30	Simple & efficient	Low image quality	Basic GAN implementation for generative tasks
DCGAN	45.0	28	Improved stabality	Limited scalability	Improved training stability for image generation
CycleGAN	32.0	25	Effective for style transfer	High training complexity	Style transferred between unpaired image sets
StyleGAN2	12.4	22	High resolution outputs	Requires extensive training	High quality image generation with complex architectures
StyleGAN3	8.2	20	Improved texture and artifacts	Computationally intensive	Cutting-edge advancements in texture generation and artifact reduction
Pix2Pix	28.5	24	Works well on paired data	Needs paired datasets	Paired image-to-image translation (e.g., sketch to photo)
BigGAN	14.0	18	High quality & diverse images	Requires massive compute resources	Class-conditional high-fidelity image generation
Progressive GAN	17.8	21	Stable training for large images	Slower training times	Progressive image resolution improvement
StarGAN	30.2	23	Multi-domain image translation	Weaker quality than task-specific GANs	Unified model for facial attribute editing across domains
SRGAN	26.1	27	High-res image super-resolution	May produce artifacts	Super-resolution of low-resolution images

Note: FID (Fréchet Inception Distance) measures the similarity between the generated and real image distributions in feature space — lower values indicate higher visual fidelity. Inference speed is measured in frames per second (fps), indicating how many video frames the model can process per second — higher values are preferred for real-time applications.

##### Datasets

###### FFHQ (Flickr-Faces-HQ)

FFHQ (Flickr-Faces-HQ) Springenberg et al., 2014 is a high-quality imaging dataset with 70 K images of faces by humans, and is collected from Flickr under Creative Commons license. NVIDIA released it as a metric for training and testing GANs, specifically StyleGAN. The dataset consists of high resolution (1024×1024 pixels) with diverse facial expressions, accessories, backgrounds and illuminations.

(i) Biases in FFHQ
  (a). Demographic Bias:
    Contrary to the claims of diversity, the FFHQ data is found to contain imbalances both among age, ethnicity, and gender. The bias towards lighter-skinned people and adults, particularly those with Western-focused facial features, affects the performance of the models. This results in the detection and generation models working better for some demographic groups while performing worse for others (e.g. children, elderly, people of colour).
  (b). Environmental Bias:
    Examples in FFHQ images are, for the most part, shot in good lighting conditions, in a center position of the frame, in casual or posed settings. This provides us with a defense mechanism against attacks on faces in surveillance-like conditions or under low light, leading to a domain gap between these specific environments and the deployment ones (such as CCTV or social media videos with poor illumination or occlusions).
  (c). Resolution Bias:
    All images in FFHQ are high-resolution (1024×1024), which jitter over the high-resolution vs low-resolution means that a real-world input such as a low-quality social-media video or grainy mobile-camera footage is not of practical, the low-resolution blur practical input to use for a neural network. This could induce the detection model sensitive to artifacts that are only in the high-res generated content, and hard to be applied to low-res or compressed real-content.
(ii) Ethical Considerations
  (a) Privacy and Consent:
    While FFHQ uses Creative Commons licensed images as source, many of the individuals depicted may have not given explicit consent for synthetic media and biometric model training use-cases. This poses ethical questions about privacy as well as the application of personal data in destructive applications.
  (b) Dual Use of Research:
    FFHQ-trained models have driven both detection and generation progress. Although this facilitates developments in the defence against deepfakes, it also improves the quality of synthetic media, leading to concerns about its misuse in misinformation, identity fraud and non-consensual creation of content.
  (c) Representation Fairness:
    Morally righteous datasets should strive for good representation of underrepresented communities.” Failure in doing so could allow biases to be propagated into downstream applications such as facial recognition and law enforcement tools, contributing to society-wide inequities.
(iii) Generalization Challenges
  (a) Dataset-to-Real-World Gap:
    Models trained on FFHQ or other such curated datasets often don’t generalize to real-world data, where face images have varied resolution, noise levels, compression, occlusions and/or pose. The artifacts learned by synthetic manipulations during training process may not be present under real-world manipulations.
  (b) Cross-Dataset Performance Drop:
    This has motivated a number of works that study the importance of fake detection models that are trained on the FFHQ dataset (and FFHQ generated fakes) and that perform very poorly at test-time when tested on different datasets (e.g., Wild Deepfake, Celeb-DF or user generated content) due to overfitting to FFHQ-specific features and generation methods.
  (c) Evolving Deepfake Techniques:
    Model Comp FFHQ is frequently used for training GAN-based generators (such as StyleGAN), newer-generation techniques and architectures (e.g. diffusion models) may introduce artefacts that are quite different. Consequently, models trained on fakes derived from FFHQ might fail to detect or incorrectly label newly generated synthetic data.

Although FFHQ has driven a number of advances in face synthesis and other techniques, we believe that its biases, ethical concerns, and lack of generalization highlight the importance of larger, more representative and more responsibly curated image datasets. In future, more attention should be paid to real-world benchmark datasets and fairness-aware training strategies to enable more robust and fair models for deepfake detection.

###### VoxCeleb

The VoxCeleb dataset series (VoxCeleb1 and VoxCeleb2) contains hundreds of thousands of speech-video clips extracted from YouTube interviews of celebrities. It was designed for speaker recognition, face verification, and audio-visual learning tasks. The dataset includes synchronized video and audio, making it widely used in research involving audio-driven face synthesis, speech cloning, and lip-sync deepfakes (e.g., Wav2Lip, SyncNet, and talking head generation models).

(i) Biases in VoxCeleb
  (a) Demographic Bias:
    VoxCeleb exhibits a notable celebrity-centric bias, as most subjects are public figures from Western media. This results in:
    - Overrepresentation of English speakers, particularly American or British accents.
    - Underrepresentation of non-Western ethnicities, dialects, and age groups.
    - Gender imbalance (especially in VoxCeleb1), potentially skewing model performance.
      These demographic biases can lead to uneven detection accuracy, especially when evaluating manipulated videos involving underrepresented voices or faces.
  (b) Environmental Bias:
    Most videos in VoxCeleb are professionally recorded interviews with controlled lighting, clean backgrounds, and high audio-visual clarity. This contrasts with real-world deepfake scenarios where audio and video may be noisy, occluded, off-angle, or recorded in uncontrolled environments. Models trained on VoxCeleb often perform poorly in low-quality or spontaneous video contexts.
  (c) Resolution and Format Bias:
    The dataset contains relatively high-resolution video and high-fidelity audio, which is not reflective of many deployment environments such as social media, messaging apps, or CCTV footage. Deepfake detectors may rely on high-frequency audio or visual cues absent in compressed or degraded real-world content.
(ii) Ethical Considerations
  (a) Consent and Public Availability:
    While VoxCeleb only includes data from publicly available videos (YouTube), the individuals involved did not explicitly consent to their data being used for biometric research, especially in applications involving face reenactment or speech synthesis. This raises concerns about:
    - Involuntary data usage
    - Potential misuse of celebrity likenesses
    - Privacy infringements
  (b) Dual-Use Risk:
    The dataset has facilitated development of powerful audio-visual generation models, enabling:
    - Realistic lip-sync deepfakes
    - Speech-to-video talking head generation
    - Cross-modal biometric attacks
      These tools can be misused for impersonation, misinformation, and non-consensual synthetic media, necessitating responsible disclosure and usage controls.
  (c) Reinforcing Stereotypes:
    Because the dataset emphasizes Western celebrities, models trained on VoxCeleb may reinforce stereotypes or marginalize speech and facial patterns not well-represented, impacting fairness and inclusivity.
(iii) Generalization Challenges
  (a) Cross-Domain Generalization:
    Models trained on VoxCeleb data (e.g., for deepfake detection or identity verification) often struggle to generalize to non-celebrity, in-the-wild data. Reasons include:
    - Domain shift (posed vs. spontaneous behavior)
    - Differences in speech patterns, facial expressions, and recording quality
  (b) Audio-Visual Synchronization Bias:
    Many deepfake detectors exploit temporal inconsistencies between speech and lip movements. Since VoxCeleb provides well-synchronized source material, detectors may be overly reliant on ideal sync conditions, failing in scenarios with asynchronous or partially corrupted audio-visual streams.
  (c) Dataset-Specific Artifacts:
    Synthetic data generated using VoxCeleb may contain artifacts unique to the training pipeline (e.g., those from Wav2Lip or GAN-based reenactment models). Detection models may overfit to these, making them brittle against newer or unseen generation methods (e.g., diffusion models or real-time streaming manipulations).

While VoxCeleb has been a cornerstone dataset for audio-visual research, its demographic skew, resolution constraints, and ethical gray areas limit its effectiveness for training generalized deepfake detection systems. Responsible use of VoxCeleb requires bias mitigation, complementary real-world datasets, and stronger ethical frameworks to ensure that detection models perform equitably and remain resilient against evolving threats.1.2.2.3 DeepFake Detection Challenge (DFDC) Dataset:

The DeepFake Detection Challenge (DFDC) dataset was introduced by Facebook AI along with industry and academia to facilitate the research of deepfake detection. It is composed of 100,000+ videos (real + deepfake) produced with different synthesis methods, which includes face swapping, reenactment, as well as other methods. The dataset was released in two stages: the preview dataset (December 2019), and the full dataset (early 2020) for the global DFDC competition.

(i) Biases in DFDC
  (a) Demographic Bias:
    Although DFDC tried to include a variety of protagonist(gc) in the AGNs in terms of gender, ethnicity and age, separate works have determined:
    - Disproportionate representation of some ethnic groups, namely white and non-dark skinned people.
    - Neglect of non-Western facial traits, which may cause the model to perform worse on minority groups.
    - A gender-age imbalance that would compromise the fairness of detection for older people in an identity that is non-binary.
  (b) Environmental Bias:
    DFDC videos are captured under a relatively controlled lighting environment and background with efforts to simulate variations in pose, clothing, and movements of the head. However:
    - The dataset does not contain widely varying extreme environments such as those found in users’ own video or surveillance video (e.g., outdoors, occlusion, and night).
    - Most clips are short, talking-head-style scenes, where an individual talks directly into a camera, which limits exposure to realistic scenarios with natural dynamics such as multiple faces, background noise, and motion blur.
  (c) Resolution Bias:
    The videos in DFDC are of relatively high quality (often 480p or 720p), and are not very compressed. This stands in contrast to latter day real world social media and deepfake encoded artefacts compressions downscaling and resolution restricting. DFDC trained detection models may bias high-res synthetic artifacts that are not present in low-res forgery samples.
(ii) Ethical Considerations
  (a) Conditions of Consent and Data Use:
    It consists of paid actors who have given their informed consent to use their videos in synthetic media research. This is an ethical step up compared to other datasets such as Celeb-DF or FaceForensics++ which frequently employ content without appropriate approval.
    However, The deepfake generation process to produce fakes on these videos is not fully documented which inhibit transparency for reproducibility and potential inspection of training bias. The external validity of such actors is still in doubt, since the actors knew that they were under recording, causing to behave in an unnatural manner rather than a natural way.
  (b) Dual-Use Risks:
    While these high-quality deepfakes were intended for researchers looking to develop new detection technologies, they can nevertheless be re purposed to train generation models as the arms race between authoring and detection of fake content continues. This underscores the persistent ethical balance involved in sharing such potent datasets.
  (c) *Re*-identification Risks:
    Even when the actors consent, addition of audio and video to large public datasets could still jeopardize re-identification of the people involved, especially when it can be used alongside other open-source intelligence (OSINT) software.
(iii) Generalization Challenges
  (a) Domain Gap with the Real-World Deepfakes:
    Models pre-trained on DFDC have a good performance on test split of DFDC, however, they often fail to generalize on:
    - Different angles lighting movement camera and compression social media filmed videos.
    - Novel deepfake methods (e.g., diffusion-based or real-time streaming fakes) unobserved at DFDC training time.
  (b) Overfitting to DFDC Artifacts:
    Due to the fact that DFDC incorporates a finite sampling of deepfake generation pipelines, it is possible that the detection models overfit over certain type of synthetic artifacts (e.g., blending errors and warping), present in this kind of pipelines. With advances in learning for new generation methods, the existence of these artifacts are harder to detect and yield models performing poorly on out-of-sample manipulations.
  (c) Intra-dataset Similarity:
    Many DFDC videos share the same actors between different fakes (and including a few real samples), therefore, models tend to memorize actors-specific cues rather than learning general features of manipulated data. This lack of generality is not very robust in novel situations.

With scale, diversity, and ethical oversight, the DFDC dataset represents a huge step toward fighting deepfakes. However, in spite of its strengths, DFDC has some limitations such as demographic biases, controlled environments, and the lack of recent generation techniques. The poor generalization of detection models in the wild (where videos from different sources differ in content quality and manipulation complexity) is a result of these limitations. In the future, it is important to investigate cross-dataset training, adversarial robustness, bias mitigation, and the kind to construct a real-world-ready detection system.There are more datasets available in the market, as shown in Table 2 [16], [25], [26], [27], [32], [33], [35], [40], [52], [66], [67] below.

**Table 2:** Commonly used Datasets.

Dataset	Size	Content	Key Features	Purpose
FFHQ (Flickr-Faces-HQ)	Large dataset	High-quality human face images	Used for training GANs	Face synthesis applications
VoxCeleb	Large dataset	Video and audio clips of celebrities	Synchronized audio-visual data	Lip-sync and speech synthesis systems
FaceForensics++	1000+ videos	Real and manipulated	High-quality fakes, multiple codecs	Benchmark for static videos
DeepfakeDetection	3600 videos	Manipulated videos	Realistic manipulations, varied sources	Training large models
Celeb-DF (v2)	5639 videos	Celebrity faces	High visual quality, fewer artifacts	Real-world applications
DFDC (Deepfake Detection Challenge)	128,000+ videos	Real and fake videos	Diverse subjects, real-world scenarios	Model benchmarking
DeeperForensics-1.0	10,000 videos	Challenging manipulations	Variability in compression levels	General robustness testing
Kaggle Deepfake Dataset	50,000+ videos	Public deepfake competition dataset	Mix of real and AI-generated faces	Community-driven detection model training
WildDeepfake	3000+ videos	Real-world deepfakes	Collected from the internet, in-the-wild scenarios	Enhancing detection in uncontrolled settings
DF-TIMIT	620 videos	Controlled synthetic videos	Based on TIMIT speech corpus	Face-swapping model evaluation
FaceSwap-DB	10,000+ images	Face swap manipulations	Controlled face swapping, alignment annotations	Face replacement detection tasks
SynthesEyes	500,000+ images	Synthetic eye region images	High precision eye images with labels	Eye-tracking and gaze estimation
Google Deepfake Dataset	∼3000 videos	AI-generated faces	Contributed by Google AI	Deepfake detection system evaluation

Deepfake technology's widespread availability allowed for more creative applications in marketing, education, and entertainment by democratizing public access.

**Fig. 2:**
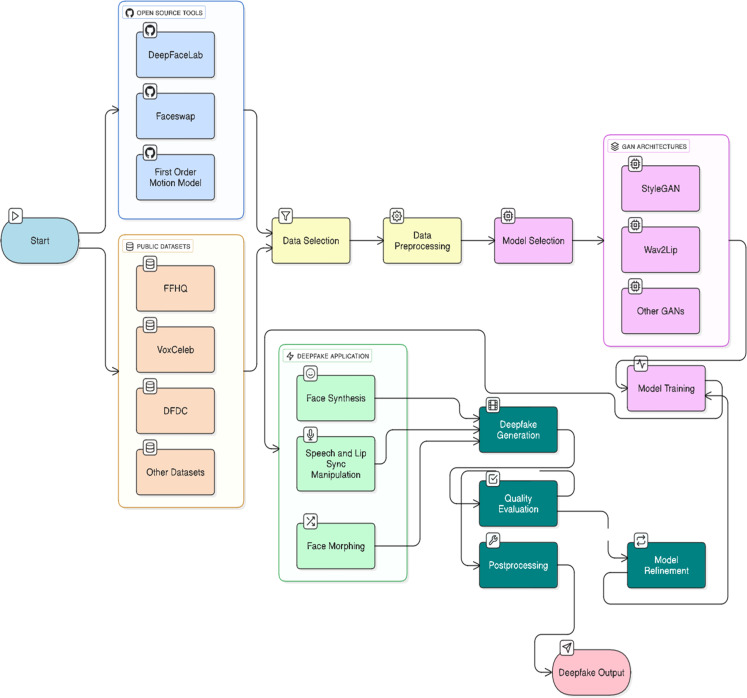
Workflow of deepfake creation using open-source tools, public datasets, and GAN architectures.

Fig. 2 illustrates the entire process of creating a deepfake, starting with open-source software and datasets and ending with the selection and fine-tuning of a GAN-based model [62]. However, it comes with problems that put its security at risk because there are more opportunities for dubious misuse, such as the spread of false information, identity theft, and cyberbullying.

Deepfake production techniques have revolutionized the field of synthetic media. They are driven by advancements in GANs and supported by publicly available software tools and datasets. The possibilities for creativity and inventiveness are as exciting as technological advancements, but equally significant are the ethical and societal issues they raise. These problems will require a multifaceted approach that includes technological protections, legal procedures, and public education initiatives to understand the processes and components underlying the creation of deepfakes.

### State-of-the-Art detection approaches

Artificial intelligence and deep learning in particular, advanced at an extraordinary pace that had revolutionized the detection of manipulations [10] within images and videos. With this progress, higher expectations in fake content creation followed with an ever-increasing complexity, therefore causing massive obstacles for establishing true authenticity and integrity for the visual data [9].

These challenges are accompanied by new detection approaches utilizing the same technology in order to refine our capability in detecting manipulations [23] in a more accurate manner. These detection approaches include AI-based approaches, deep learning-based ones, as well as biometry-oriented approaches. These approaches, in addition to paying attention on detecting manipulations in images and video, work on combating new threats in the form of spoofing in biometry systems [24].

**Fig. 3:**
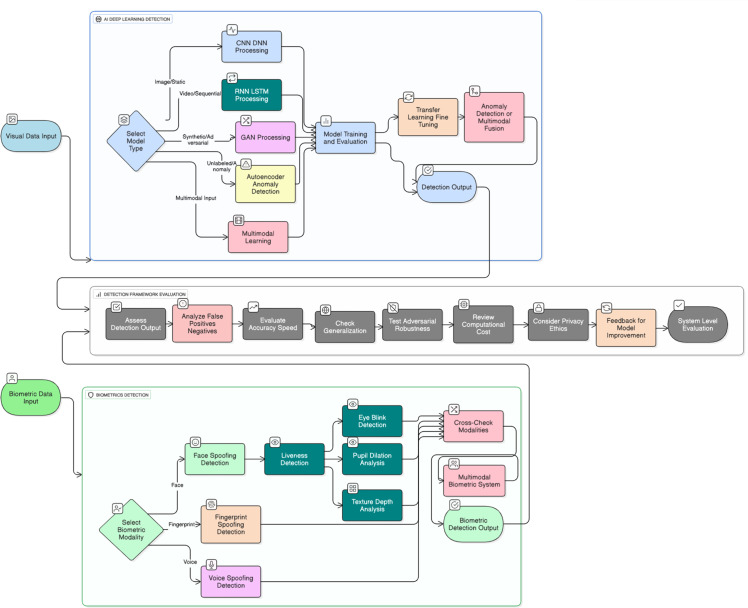
Workflow of state-of-the-art deepfake detection approaches combining AI-based methods and biometric-focused techniques.

Fig. 3 Shows the end-to-end detection system, from input data and model choice in AI-driven detection (CNN, RNN, GAN, anomaly detection, and multimodal learning), all the way through the system evaluation phases (accuracy, generalization, adversarial robustness, computational cost, and ethics), and then biometric-specific detection procedures, namely face, voice, and fingerprint spoofing detection, and liveness detection in the form of eye blink, dilated pupils, and texture analysis, all the way up through a final end-to-end detection result.

#### AI and deep learning methods for identifying image and video manipulations

The detection of manipulations in images and videos has become central to the task of dealing with this complex problem [6]. Traditional approaches, such as digital forensics [102], focus on the physical properties of noise patterns or compression artifacts in images. Modern manipulations are now able to bypass these conventional methods, and hence AI-based methods have become essential [10]. Table 3 summarizes the primary AI and deep learning-based detection techniques, their targeted manipulation types, example applications, strengths, limitations, and key references, providing a comparative overview of state-of-the-art methods.

**Table 3:** Mapping of Deepfake Detection Techniques to Manipulation Types.

Detection Technique	Manipulation Type	Example Applications	Capabilities	Limitations	References
CNN-Based Models (e.g., XceptionNet, EfficientNet)	Image / Video	Face swap, facial reenactment	High accuracy in detecting spatial artifacts	Sensitive to compression and resolution	[39], [53], [56], [76]
RNN / LSTM-Based Models	Video	Temporal forgery detection	Captures motion-based artifacts	Slow inference; high computational cost	[77]
Transformer-Based Models (ViT, TimeSformer)	Video	Spatiotemporal deepfake detection	Strong performance with sufficient data	Resource-intensive; unsuitable for real-time	[78], [79], [103], [104], [105]
Audio Spectrogram CNNs	Audio	Voice cloning, speech synthesis	Detects waveform and frequency anomalies	Less robust to high-quality fakes with clean audio	[80]
Lip-Sync Inconsistency Models (e.g., SyncNet)	Audio + Video	Lip-sync deepfake detection	Effective at detecting mouth-speech mismatches	May fail with synchronized manipulations	[81], [82]
Multimodal Fusion Models	Audio + Video + Text	Simultaneous detection across modalities	High robustness to diverse manipulations	Requires alignment and synchronization	[83], [84]
Biometric-Specific Detection (e.g., heart rate, iris, gait)	Biometric (Face, Voice)	Liveness detection, spoofing prevention	Exploits physiological signals (e.g., PPG, blink rate)	Input quality sensitive; subject-dependent	[85]
Autoencoder / Anomaly Detection Models	Image / Video / Audio	Zero-shot or few-shot deepfake detection	Detects previously unseen manipulations	Threshold sensitivity; lower precision	[57], [61], [86]
Metadata & Compression Analysis	Image / Video	Passive forensics (EXIF, codec artifacts)	Lightweight, interpretable	Bypassed with re-encoding or manipulation cleanup	[87]
Ensemble / Hybrid Models	Cross-modal (Image/Video/Audio)	Robust detection pipelines	Combines strengths of multiple techniques	Complex design; resource-heavy	[64], [66], [69], [77], [85]

##### Deep neural networks (DNNs) and convolutional neural networks (CNNs) [64]

Generally, these networks are particularly effective in detecting visual anomalies in manipulated images or videos. Spatial hierarchies in visual data make CNNs particularly useful in distinguishing between real and manipulated images [68]. Large datasets of authentic and manipulated images must be trained on to teach CNNs the ability to automatically detect inconsistencies like unnatural lighting, edge artifacts, or pixel-level changes.

##### Generative adversarial networks

GANs are deep learning that pertains to training two networks. These are generator and discriminator together. The primary task of a generator is creating fake images while the discriminator determines real from the fake images [65]. Gradually, the discriminator learns how to pick subtle manipulations very well. GANs can be used to generate fake content (for research purposes, which involves creating synthetic training datasets) as well as detect such manipulations.

2.1.3 Recurrent Neural Networks (RNNs) and Long Short-Term Memory (LSTM). In video manipulation detection, RNNs [63] and LSTMs are important for their ability to process sequential data. For instance, an RNN can follow up on inconsistencies in time across videos such as unnatural movement patterns, audio mismatch, or even abrupt changes in the sequence of frames that often reflect tampering [15]. It can also aid in deepfake video detection, which might help to catch up with the tiny inconsistencies of the facial expression, voice sync, or other movements that do not exist in real life.

##### Transfer learning and pretrained models

Deep learning has the habit of using transfer learning, in which a model is pretrained on a large, diverse dataset and adapted to a smaller, specific dataset for a particular task. In the context of manipulation detection, it can be applied to adapt general object recognition models to recognize specific manipulation features, which can dramatically improve efficiency and accuracy [14]. Models such as VGGNet, ResNet, and InceptionNet have been pre-trained on large image datasets and have been impressive when fine-tuned for manipulation detection tasks.

##### Autoencoders and anomaly detection

Autoencoders compress and reconstruct the data in an attempt to reconstruct the original. Detection of any manipulations can easily be performed using such an autoencoder because manipulations will always cause differences in the relationships between pixels, and this difference can easily be found [16] to indicate an anomaly. Algorithms applying anomaly detection on latent space can easily point out differences that indicate manipulation.

##### Multimodal learning. multimodal learning is another cutting-edge technique

deep learning models are fed in real time different kinds of data at once-visual, audio, text-and so forth. In video manipulations, like deepfakes [40], a multimodal system could analyze simultaneously both the visual frames and the accompanying audio track. Such a system would alert one to inconsistencies in lip movement with speech. Similarly, multimodal models detect inconsistencies between body movements and background lighting in manipulated video content.

#### Biometrics-Focused detection techniques against spoofing attacks

Biometric systems such as facial recognition, fingerprint scanning, and voice recognition are increasingly used for security purposes [38]. However, with the widespread use of such systems, the sophistication of spoofing attacks - where an attacker presents fake biometric data - has increased. Thus, there is a great need for biometric-focused detection techniques to identify such spoofing attempts.

##### Face recognition spoofing detection

Face recognition systems are particularly vulnerable to spoofing attacks where attackers use photographs, videos, or 3D models of a target’s face to gain unauthorized access. Anti-spoofing techniques based on deep learning have been developed to address this issue [36]. These techniques focus on detecting signs of a fake face, such as inconsistent lighting, reflections, or lack of natural depth. These are the most used CNN-based approaches for spatial features analysis in faces [37], with texture and geometric discrepancies being an indication of spoofing attacks.

##### Liveness detection

One of the strongest anti-spoofing is liveness detection, which validates that the presented biometric sample to the system is from an alive person instead of a photograph or video static image. Techniques for liveness detection can work on multiple cues such as;

##### Eye movement or blink detection

Detection of eye movement or blinking behavior that cannot be mimicked through static images or video [19].

a. Pupil dilation analysis: The variation in pupil size when light is reflected on it cannot be replicated by the spoofing device [51].
b. Texture and depth analysis: Deep learning models can analyze textures and depth information to differentiate between live faces and fakes.

##### Fingerprint spoofing detection

Like fingerprint-based recognition systems are susceptible to spoofing, where attackers use molds or fake fingerprints against the sensor [58]. Identification of a fake fingerprint is a matter of advanced pattern recognition techniques. Deep learning models can be trained over large datasets of real and spoofed fingerprints to learn the subtle differences from the genuine ridges, pores, and minutiae points of the real fingerprint [10] versus those of the spoofed fingerprint.

##### Voice spoofing detection

When voice recognition systems are spoofed with recorded or synthesized voices, bypassing the security mechanism, the underlying problem is a challenge to these systems. Techniques such as spectrogram analysis convert sound waves into a visual representation that could help detect fake voices [50], [55] by identifying inconsistencies in the frequency spectrum. Another feature with deep learning is the ability of models to analyze and identify features from prosody such as rhythm and pitch of speech together with formant, which might indicate spoofing.

##### Multimodal biometric systems

**Fig. 4:**
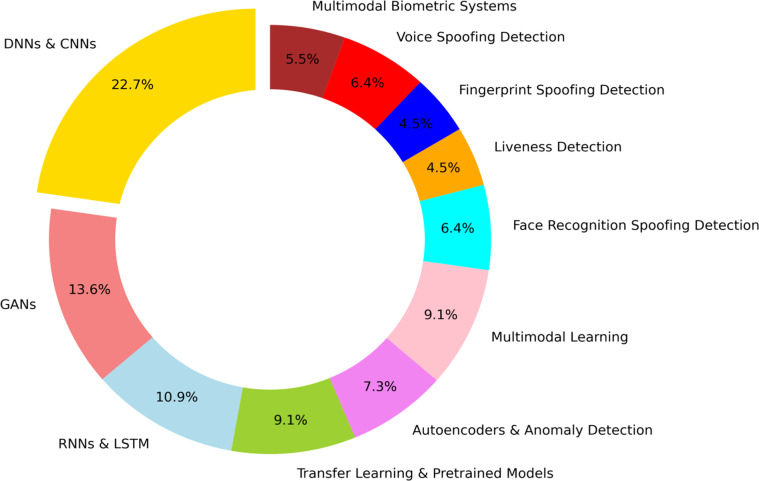
xxxx.

For higher robustness, multimodal biometric systems are developed by integrating various biometric traits, for example, facial recognition and voice recognition. With the help of complementary strengths of different biometric modalities, these systems can detect spoofing attempts [54] that may be successful against a single modality. Machine learning algorithms can analyze patterns across different modalities and find inconsistencies that may indicate an attack. Fig. 4 Shows the realistic percentages of how above mentioned technologies contribute to overall detection of deepfakes and biometrics,

##### Complementary defenses against biometric spoofing

In the face of increasingly elaborate deepfake-based spoofing attacks on biometric systems, a defense in depth is necessary. The traditional approaches to detection and liveness detection are important, but the addition of complementary methods provides a substantial increase in robustness. The other important, synergistic defense mechanisms are:

(i) Continuous Authentication
  Unlike a traditional one-time biometric verification at login, continuous authentication is concerned with assuring the identity of the user throughout an active session, by means of behavior and context biometrics. This technique continuously assures the user of the identity through:
  • Recognition of Gait from smartphone sensors or CCTV.
  • Keystroke dynamics (typist rhythm and speed).
  • Mouse movement patterns.
  • Facial micro-expressions and gaze monitoring with front-facing camera-based devices.
    Continuous authentication systems are able to identify and stop anomalous behaviour even after a login and as such it's extremely hard for someone using spoofed biometric to maintain access, undetected. This particularly convenient in high security cases, or long sessions i.e. banking or remote work.
(ii) Multi-Factor Authentication (MFA)
  Multi-factor also refers to types beyond the biometric modality. A typical MFA system combines:
  • Something the user remembers (PIN, password).
  • What the user has (eg a smartphone or hardware token)
  • Something the user has (such as a fingerprint or face or voice)
  • Something the user is (for example fingerprints or face or voice).
    When a biometric spoof attack puts biometric data at risk, MFA can still prevent unauthorized access by simply not allowing all reveals of factors to succeed. For example, with facial deepfake feel, and (the) camera has been cheated, the manner in which a time-based OTP (One-Time Password) was transmitted to registered device and/ or display the password to user is different.
(iii) Advanced Liveness Detection Techniques
  Liveness detection confirms that the biometric input originates from a living subject now—it is not a photograph, video, or deepfake. More advanced techniques include:
  • 3D Depth Sensing: Measures depth for features using structured light or time of flight sensors. Flat images and video struggle to model three-dimensional ridges.
  • Thermal Imaging: Shows you real skin temperature versus nonliving surfaces. Spoofing with masks or screens does not re-create heat distribution.
  • Micro-Motion Analysis: Monitors involuntary human traits such as eye micro-movements, changes in skin texture and minute muscle movements, which are difficult to copy in deepfakes.
  • Reactive Response Tasks: Compels the user to react to random stimuli such as rhythmic blinking, head turning, or saying random words, rendering prerecorded media ineffective.
    These methods form strong barriers against static / pre-computed deepfakes.
(iv) Multimodal Biometric Fusion
  Multimodal systems, as opposed to unimodal, do not rely on a single biometric characteristic, but on at least two biometric inputs – e.g.,
  • Face + Voice
  • Fingerprint + Iris
  • Voice + Gait
    This is how it would become near-impossible for attackers to produce high-quality deepfakes on diverse modalities. It is possible, for example, to create a spoofed face using GANs (Generative Adversarial Networks), but it becomes exponentially harder to simultaneously syn- thesize real-time speech and gait of the person.
    Besides, the fusion mechanism applied here is multi-level:
  • Raw datalevel fusion: Integration of raw data streams.
  • Feature Fusion at the Feature Level: Extract and Combine Characteristics.
  • Decision level fusion: Combination of independent decisions from each modality.
(v) AI-Driven Behavioral and Anomaly Analysis
  Adversary detection is especially challenging because adversaries attempt to evade detection by acting like an honest user. Machine learning models, trained only on benign user behavior, can detect faint line profiles emerging from imposters. For example:
  • A voice cadence and emotion analysis to detect robotic or overly smoothed artificial speech.
  • Facial emotion detection can identify the out-of-place or fixed expressions in video deepfakes.
  • User context modeling: Fuses data such as location, time of access, and type of device to identify anomalies (a login attempt from a new location with the same biometric details).
    Models of this kind become stronger and stronger as they learn over time and provide an adaptive barrier against new spoofing attacks.
(vi) Biometric Templates That Preserve Privacy
  Biometric data must be kept secure. In contrast to passwords, conventional systems preserve raw or hash-based biometric templates, which are irreplaceable in the event of compromise. More recent techniques include:
  • Revocable biometrics: Reconstruct biometric transformation using mathematical functions that cannot be reversed, allowing corrupted templates to be "revoked" and reissued.
    Biometric matching within an encrypted domain is made possible by homomorphic encryption, which conceals the raw data.
  • A private/federated biometric submission system Reduce the surface area and avoid centralizing the store and authentication on the device (Secure Enclave Apple).

These practices can help prevent dataset leaks and stop synthesis of fake media involving stolen biometric data. Table 4 summarizes key privacy-preserving defense mechanisms, the targeted threats they address, and their advantages in securing biometric systems.

**Table 4:** Biometric defense mechanisms against spoofing and data theft.

Defense Mechanism	Targeted Threat	Key Advantage
Continuous Authentication	Session hijacking, delayed spoofing	Monitors user throughout interaction
Multi-Factor Authentication	Single-point spoofing	Adds independent security layers
Advanced Liveness Detection	Static video/photo spoofing	Validates presence of a live individual
Multimodal Biometric Fusion	Modality-specific spoofing	Combines traits for stronger verification
AI-Based Anomaly Detection	Synthetic behavior emulation	Detects behavioral irregularities
Cancelable Biometric Templates	Biometric data theft	Enables revocation and privacy preservation

#### Implementation and reproducibility

Reproducibility is crucial in the investigation of deepfake generation and detection. This article reports usual hyperparameter settings, preprocessing pipelines, model-specific concerns, and open-source material from current state-of-the-art literature.

##### Hyperparameter configurations

Generation and detection deep learning techniques have a number of hyperparameters in common that are typically fine-tuned. Table 1 shows common values used in GANs, CNNs, RNNs, and Transformers. The table highlights ,

• Learning Rate → How fast the model updates weights.
• Batch Size → Number of samples per training iteration.
• Optimizer → Algorithm used for weight updates.
• Epochs → How many times the full dataset is seen during training.
• Dropout → Regularization to prevent overfitting.
• Loss Function → What the model tries to minimize (differs by task).
• Weight Initialization → How starting weights are set before training.

along with remarks on best practices. This consolidated overview serves as a quick reference for setting up experiments and ensuring comparability across studies.

Table 5, Table 6, Table 7.

**Table 5:** Typical Hyperparameters in Deepfake Models.

Parameter	GANs (StyleGAN, BigGAN)	CNNs (Xception, EfficientNet)	RNNs/LSTMs	Transformers (ViT, TimeSformer)	Remark
Learning Rate	1e-4 (with decay)	1e-4 (Adam)	5e-4 → 1e-5 (decay)	5e-5 (AdamW)	Transformers benefit from AdamW with weight decay
Batch Size	16–32	32–64	16–32	8–16 (memory heavy)	Video models require smaller batch sizes
Optimizer	Adam (β1=0.5, β2=0.999)	Adam (β1=0.9, β2=0.999)	Adam/SGD	AdamW	GANs use β1=0.5 for stability
Epochs	100–300	50–100	30–50	30–50	Early stopping prevents overfitting
Dropout	0.3 (discriminator only)	0.3–0.5	0.2–0.5	0.1–0.3	Transformers need less dropout
Loss Function	Adversarial + Perceptual Loss	Binary Cross-Entropy / Focal	BCE / Hinge Loss	Cross-Entropy / Contrastive Loss	Focal loss helps with class imbalance
Weight Initialization	He Normal (conv layers)	Xavier Init	Orthogonal	Pretrained ImageNet Weights	Transfer learning is standard

Note: 1e-4 (with decay) refers to a learning rate of 0.0001 that is not fixed, but gradually reduced (decayed) during training.

**Table 6:** Summarizes the performance of prominent detection methods based on metrics.

Method	Accuracy ( %)	Inference Speed (fps)	Strengths	Weaknesses/Limitations in Streaming Context	Use Case	Key Features	Real Time Capable
Handcrafted Features	72.5	35	Low computational cost	Limited generalizability	Lightweight applications	Relies on predefined visual patterns	Yes
CNN-Based Detection	88.0	30	High accuracy for static images	Struggles with temporal consistency	Image-based deepfake detection	Explores spatial features effectively	Yes
RNN-Based Detection	85.5	25	Good for video sequences	High computational complexity	Video sequence analysis	Focuses on temporal correlations	Yes(Limited)
Transformer-Based Detection	92.3	20	Robust to multiple manipulations	Requires large datasets	Advanced manipulation detection	Captures both spatial and temporal patterns	No
Hybrid Methods	95.0	22	Combines strengths of multiple methods	High computational and training cost	Comprehensive detection across modalities	Integrates CNN, RNN, and transformer approaches	No
Autoencoder Anomaly Detection	80.0	32	Unsupervised; no labeled fakes needed	High false-positive rate	Unlabeled data environments	Learns reconstruction errors to detect anomalies	Yes
XceptionNet-Based	90.2	28	Excellent fine-grained feature extraction	Large model size	Image-only deepfake detection	Uses depthwise separable convolutions	Yes
Capsule Network Detection	87.5	24	Robust to spatial transformations	Slower convergence	Small datasets	Models spatial hierarchies using capsules	Yes(BorderLine)
Siamese Network Detection	86.8	26	Learns similarity, supports few-shot learning	Requires careful pair selection	Cross-dataset detection	Compares real vs. fake samples with twin networks	Yes
Multimodal Fusion Detection	93.1	18	Uses audio-visual data for better accuracy	Requires careful synchronization	Audio-visual deepfake detection	Fuses CNN (video) and RNN (audio) streams	No

**Table 7:** Computational Cost Implications of Deepfake Detection Models.

Model Type	Accuracy ( %)	Inference Speed (FPS)	Computational Cost	Deployment Feasibility	Use Case Suitability
CNN-Based (e.g., XceptionNet)	∼88–90 %	∼28–30	Moderate	Medium (mobile/cloud platforms)	Image-based deepfake detection
RNN/LSTM-Based	∼85–87 %	∼20–25	High	Low (requires powerful backend)	Temporal/spatiotemporal video analysis
Transformer-Based	∼92–93 %	∼18–22	Very High	Low (cloud or data center only)	Advanced manipulation detection
Multimodal Fusion	∼93–95 %	∼18–20	Very High	Low (backend infrastructure only)	Audio-visual deepfake detection
Autoencoder/Anomaly Detection	∼80 %	∼30–32	Low to Moderate	Medium (adaptive for edge use)	Unsupervised/novelty-based fake detection
Hybrid Methods (CNN + RNN + Transformers)	∼94–95 %	∼20–22	Very High	Low (specialized infrastructure)	High-accuracy, cross-modal detection systems

FPS (Frames Per Second) is estimated for a frame resolution of 224×224 on standard GPU.

Computational cost includes the costs of training, inference, memory, and energy.

We classify feasibility according to the suitability for mobile, edge or massive cloud environments.

##### Dataset preprocessing pipelines

Reproducibility is highly dependent on consistent data preprocessing.

• Image-based datasets (FFHQ, CelebA, FaceForensics++):
  • Face detection: MTCNN or Dlib for alignment.
  • Resize: 224×224 (CNN-based), 299×299 (Inception-based).
  • Normalization: pixel values scaled to [0,1] or standardized with ImageNet mean/variance.
  • Augmentations: flipping, Gaussian noise, JPEG compression, random cropping.
• Video datasets (DFDC, Celeb-DF, WildDeepfake):
  • Frame extraction at 25–30 fps.
  • Face-region cropping + resizing to 224×224.
  • Temporal sampling: every nth frame or sliding window sequences (16–32 frames).
  • Compression simulation: apply H.264/MP4 compression to mimic real-world uploads.
• Audio datasets (VoxCeleb, ASVspoof, TIMIT):
  • Convert raw audio to Mel-spectrograms (hop length=256, FFT=1024).
  • Extract MFCCs (20–40 coefficients) for CNN input.
  • Normalize to zero mean, unit variance.
  • Augment with noise injection, reverberation, speed perturbation.
• Implementation-level clarity (preprocessing, pseudocode):

Algorithm: End-to-End Deepfake Detection Workflow

Input: Video dataset D

Output: Real/Fake classification results

(i) Data Preprocessing
  For each video v ∈ D:
  (a) Extract frames at 25–30 fps
  (b) Detect and align faces using MTCNN or Dlib
  (c) Resize frames to 224×224 (CNN) or 299×299 (Inception)
  (d) Normalize pixel values to [0,1]
  (e) Apply augmentations: flipping, Gaussian noise, JPEG compression
(ii) Feature Extraction
  - Use pretrained CNN (XceptionNet / EfficientNet) for frame-level features
  - Optionally extract audio features (MFCCs, spectrograms) for multimodal fusion
(iii) Temporal Modeling
  - Feed sequential features into LSTM/Transformer (16–32 frame windows)
(iv) Classification
  - Apply Softmax / Binary Cross-Entropy loss for Real vs. Fake decision
(v) Evaluation
  - Report Accuracy, AUC, F1-score
  - Perform cross-dataset testing (e.g., train on DFDC, test on WildDeepfake)

##### Model-Specific considerations

• GANs:
  • Training stability requires gradient penalty (WGAN-GP).
  • Adaptive discriminator augmentation (ADA) improves generalization.
  • StyleGAN3 uses mapping networks with controlled latent vectors.
• CNNs (e.g., XceptionNet, EfficientNet):
  • Pretrained on ImageNet, fine-tuned on deepfake datasets.
  • Focal loss combats class imbalance in detection datasets.
  • Works best with JPEG compression augmentation to simulate social media uploads.
• RNNs / LSTMs:
  • Sequence length = 16–32 frames (longer sequences increase memory cost).
  • Bidirectional LSTMs improve temporal artifact detection.
  • Often combined with CNN feature extractors (CNN+LSTM hybrid).
• Transformers (ViT, TimeSformer):
  • Require large datasets (DFDC, Celeb-DF) or pretraining.
  • AdamW optimizer with learning rate warmup improves convergence.
  • Strong performance but limited for edge/mobile deployment due to high FLOPs.

##### Reproducibility practices

To ensure transparency and ease of replication:

• Code Release: Provide training + inference scripts (GitHub/Zenodo).
• Pretrained Weights: Host models on HuggingFace Hub or PyTorch Hub.
• Random Seeds: Fix seeds for NumPy, TensorFlow, and PyTorch.
• Hardware Documentation: Report GPU model, memory, training time.
• Evaluation Protocols: Clearly define train/val/test splits (avoid dataset leakage).
• Performance Metrics: Accuracy, AUC, EER (Equal Error Rate), F1-score.
• Cross-Dataset Testing: Report performance on multiple benchmarks (DFDC → WildDeepfake).

##### Resource availability

• Generation: StyleGAN2/3, DeepFaceLab, First Order Motion Model.
• Detection:XceptionNet, EfficientNet, TimeSformer, Vision Transformer (ViT).
• Multimodal:SyncNet, Wav2Lip.
• Datasets: FFHQ, DFDC, Celeb-DF, VoxCeleb, WildDeepfake, ASVspoof.

#### Comparison of detection frameworks and their limitations

Although the above discussed techniques have been found to be promising, they do have limitations that must be weighed in discussing evaluation of detection frameworks [6].

##### Accuracy and false positives

One of the most significant challenges with AI-based detection models is balancing accuracy with false positives. Models that are overly sensitive may flag legitimate content as manipulated, while less sensitive models might miss subtle manipulations. This trade-off can be particularly critical in real-world applications, such as legal and security systems, where false positives can lead to severe consequences.

False positives, false negatives, and other real-world forensic failures

  (a) False Positives and False Negatives:
    In the context of deepfake detection, false positive (FP) refers to the case where real media is treated as fake, and false negative (FN) vice versa. Both are important depending on the application:
    • False Positives can result in unwarranted censorship, loss of trust and damage to reputation if genuine video is wrongly classified.
    • False Negatives, far more severely, can facilitate misinformation, identity fraud and biometric spoofing, particularly when detection cannot detect high-quality or unseen deepfakes.
  (b) Sources of False Positives
    • Compression Artifacts: Videos with com- pression artifacts (e.g., low resolution or highly compressed real video such as the ones from CCTV or social media) may look manipulated due to blocky distortions, leading to FPs.
    • Out-of-Distribution Data: Models trained on control datasets (e.g., FFHQ, DFDC), may mistakenly predict images from other domains (e.g., surveillance feeds, live streams) due to environmental conflicts.
    • Facial Occlusion and Makeup -The real-world variability, e.g., heavy makeup, occlusions (scarves, glasses), or cosmetic surgery, can significantly change facial appearance enough to deceive detection systems.
  (c) Sources of False Negatives
    • New Generation Techniques: Several detection systems are made to identify specific manipulation artifacts, such as blending artifacts and face deformation. Such cues are typically avoided by deepfakes created using diffusion models or detectors adversarially trained [59] against GANs.
    • Cross-dataset generalization problems: Due to domain and demographic shifts that result in FNs, a model trained solely on DFDC or FaceForensics++ might not be able to generalize to fakes from datasets like Celeb-DF or WildDeepfake.
    • Temporal Smoothing in Video: In order to eliminate temporally-based artifacts that detection systems used to pick up, rendering techniques have developed to enforce temporal consistency and minimize jitter across frames.
  (d) Real-World Forensic Failures
    • The 2019 Ali Bongo Incident Following his protracted absence due to poor health, a video of President Bongo surfaced in 2019. The idea that the video appeared to be a deepfake was widely circulated online by people using model guesswork and other methods. An attempted military takeover and significant political turmoil resulted from this, underscoring the possibility of misclassification as false positive even in cases where the confusion matrix is inconclusive.
    • Indian Political Speech (2020): A video which purported to show a Delhi BJP leader speaking a number of different languages was widely circulated. Validated as a deepfake by researchers soon after, it still wasn’t detected at first by social media platforms, calling attention to both the dangers of false negatives in real-world deployments, and the slowness of at-scale detection.
    • Biometric Spoofing Attacks: In the financial and access control domain, false negatives, although not occurring frame wise, have been used by attackers to defeat facial recognition with the help of deepfake videos [42], e.g., replay attacks]. "Insecureness" of the liveness detection and heavy dependence only on face images have compromised these so called secure systems.

##### Generalization across datasets

Most of the detection models are trained on particular datasets. That means, sometimes they get challenged when a new type of manipulation is applied, or if manipulated data originates from a different source. For example, deep learning model that was trained with one set of deepfake videos will not work properly with another video generated using some new techniques and advanced technologies. Detection systems must generalize well to many different types of manipulated content [10].

##### Adversarial attacks

Deep learning models are vulnerable to adversarial attacks. Adversarial attacks involve adding small, intentional perturbations to input data, such as images or videos, to deceive the model into making incorrect predictions.

For example, an attacker can manipulate a forged picture in a way that it will be difficult for a CNN to mark as a fake. This is extremely risky for the future sustainment of detection frameworks as new ways of executing undetectable attacks will not stop for some time.

##### Computational expenses and real-time

Deep learning algorithms, and AI algorithms in general, especially those that are used to identify image and video tampering, are computationally expensive.

This could be an issue in real-time applications, like social media or video streaming, where the content must be quickly recognized as tampered with. The scalability of such models may also be constrained by the computational cost of handling large data, particularly in low-end settings.

##### Privacy and ethical concerns

The detection methods raise the most ethical and privacy concerns, especially with regard to biometric platforms [8]. The boundary between enhancing security and safeguarding user privacy is very thin. For example, using biometric information for spoofing attempts or liveness checks may result in privacy violations or data leaks. To prevent misuse, biometric data collection and processing must be governed by strong privacy laws and controls.

Thanks to developments in deep learning and artificial intelligence, it is now easier to authenticate image, video, or biometric device spoofing and manipulations. In the meantime, there are still a number of issues that need to be fixed, such as avoiding adversarial attacks, reducing the number of false positives, and generalizing the model. Biometrics-specific multimodal environment detection techniques are also capable of thwarting sophisticated spoofing attack techniques. Research on detection systems must overcome these constraints without compromising computation efficiency, security, or privacy.

To evaluate the potential applicability of the proposed models in real-time environments such as social media platforms or video streaming contexts, we analyzed inference speed (in fps) across models. Real-time video typically requires inference speed of ≥24 fps to ensure smooth frame-wise analysis. Among the models tested, Vanilla GAN achieved 30 fps, suggesting its feasibility for near real-time detection. However, DCGAN and CycleGAN showed slightly lower speeds (28 fps and 25 fps, respectively), which may cause latency or frame drop in high-resolution or live streaming scenarios. Moreover, none of the models currently include live input buffering or stream parsing modules, which are essential for integration with platforms like YouTube Live or Instagram. Therefore, while the models demonstrate promising processing speed, additional engineering efforts and system-level optimizations are required for robust deployment in real-time social media applications.

#### Practical scalability

##### Challenges

• Explosion of video content: YouTube alone processes 500+ hours of video per minute; running heavy detection pipelines on all content is infeasible.
• Transformer-based models (ViT, TimeSformer, Swin, multimodal transformers) achieve state-of-the-art accuracy but often run at ∼18–22 fps on high-end GPUs, which is below real-time requirements (≥24 fps).
• Large memory footprint: Transformers require GBs of GPU VRAM, prohibitive for edge or large-scale real-time pipelines.

##### Current strategies

(i) Hierarchical (multi-stage) pipelines
  • Stage 1: Lightweight filters (blur detection, JPEG artifact checks, simple CNNs like MobileNet/EfficientNet).
  • Stage 2: Medium complexity detectors (compressed ViTs or distilled CNNs) for flagged content.
  • Stage 3: Heavy multimodal transformers only on high-value/high-risk content (political ads, financial videos).
  • This cascaded approach saves computation while preserving accuracy.
(ii) Model compression
  • Quantization (8-bit, 4-bit weights).
  • Pruning (removing redundant attention heads or layers).
  • Knowledge distillation (training a lightweight “student” from a transformer “teacher”).
  • Example: MobileViT and TinyViT achieve close-to-ViT accuracy at 1/10th compute.
(iii) Cloud–Edge hybridization
  • Lightweight models run on-device (low-latency).
  • Ambiguous samples are escalated to cloud servers with large GPUs.
  • This balances privacy, speed, and accuracy.
(iv) ROI-based processing
  • Instead of analyzing entire frames, detectors only process face regions or mouth–eye patches, cutting compute by 60–70 %.

#### Cross-Dataset robustness

##### Challenges

• Dataset bias:
  • Demographics: FFHQ and DFDC skew toward lighter-skinned, Western faces; detectors underperform on darker-skinned, elderly, or non-Western faces.
  • Environmental: Training data often has clean lighting, high resolution, while real-world content includes grainy CCTV or compressed TikTok videos.
• Technique drift: Models trained on GAN-based deepfakes (StyleGAN2, DeepFaceLab) struggle with diffusion-based fakes (Stable Diffusion, DALL·E-generated faces) that introduce different artifact patterns.
• Domain overfitting: Many detectors capture dataset-specific noise (e.g., encoding artifacts from DFDC), failing on wild user-generated deepfakes.

##### Evidence

• A CNN trained on DFDC achieves >90 % accuracy on its test set but drops to ∼60 % on WildDeepfake dataset.
• 2023–2024 studies (e.g., Raza et al., 2023; Wang et al., 2024) show transformers generalize better across datasets by capturing global spatial–temporal cues rather than pixel-level noise.

##### Current strategies

• Cross-dataset training & domain adaptation
  • Train jointly on multiple datasets (FaceForensics++, DFDC, Celeb-DF, WildDeepfake).
  • Use domain adversarial training to reduce overfitting to one dataset.
• Unsupervised & anomaly detection
  • Instead of learning artifacts, anomaly detectors model “real” distributions and flag deviations.
  • Example: Prototype-based ViTs (Aghasanli et al., 2023) detect unseen manipulations by comparing prototypes of authentic vs. fake data.
• Fairness-aware dataset construction
  • Balanced datasets (age, ethnicity, environment) are being built to minimize demographic skew.
  • E.g., ASVspoof 2024 introduced multi-condition training to boost generalization in audio deepfakes [41]
• Continuous learning
  • Models update iteratively as new manipulation methods appear (online learning, federated updates).
  • Example: Le et al., 2024 proposed transformer-based audio detectors with continuous learning.

#### Deployment limitations

(i) Adversarial Attacks
  • Evasion attacks: Small perturbations to images/videos make detectors fail while remaining imperceptible to humans.
  • Adversarial deepfakes: Generators can be adversarially trained to fool specific detectors.
  • Patch attacks: Adding stickers, glasses, or “universal patches” can bypass detection and even face recognition systems.
  • Example: Researchers showed that adversarial perturbations reduced ViT-based detection accuracy by up to 40 % in cross-dataset tests.
    - Mitigations
  • Adversarial training (inject adversarial samples during training).
  • Ensemble approaches (combine CNN + ViT + anomaly detectors).
  • Certified defenses (provable robustness, though expensive).
(ii) Resource Constraints in Edge Devices
  • Problem: Smartphones and IoT devices lack GPU/TPU compute for ViTs.
  • Transformers require hundreds of MBs of weights and high power, unsuitable for real-time edge detection.
    - Solutions
  • Use lightweight backbones (MobileViT, EfficientFormer).
  • Deploy quantized models (4–8 bit).
  • ROI-based detection (only face areas).
  • Split-compute: initial feature extraction on-device, heavy transformer inference on server.
(iii) Ethical & Privacy Concerns
  • Biometric leakage: Many detectors use liveness signals (blink detection, heart rate via PPG, pupil dilation). This risks surveillance abuse.
  • Regulatory risks: EU AI Act (2024) and India’s DPDP Act (2023) require privacy-preserving biometrics.
  • Promising directions:
    • Federated learning: models trained across devices without centralized raw data.
    • Homomorphic encryption: biometric matching without exposing raw features.
    • Cancelable biometrics: templates that can be revoked if compromised.

Summary Table

**Table:** 

Challenge	Example Issues	Research Directions (2023–2024)
Scalability	Slow transformers (<22 fps), high GPU cost	Multi-stage pipelines, model compression, ROI-based processing
Cross-dataset robustness	Demographic & domain bias, diffusion fakes	Domain adaptation, anomaly detection, fairness-aware datasets, continuous learning
Deployment limitations	Adversarial evasion, edge compute limits, privacy concerns	Adversarial training, lightweight ViTs, split-compute, federated privacy-preserving detection

#### Feasibility of real time detection and resource limitations

The real-time deepfake detection is still an open problem because of the computational burden of the current SOTA models. Recent state-of-the-art models including Transformer architectures, hybrid CNN-RNN networks, and multimodal fusion models lead to high accuracy values but they rely on heavy GPUs, large memory footprint, and long processing time per video frame or audio sample.

Image-based detection tasks can also be generally performed on relatively low-level computation power with CNN-based models (like XceptionNet) with the consideration of the equilibrium of speed and accuracy. Nevertheless, they are not yet practical for use in videos or video streams without being fine-tuned for frame sampling or being accompanied by special hardware accelerators.

Very recently, researchers have explored using RNNs and LSTMs for temporal consistency understanding on video streams, however, those models are slow in inference and memory hungry, which makes it hard to use such models in real time on edge devices (e.g., mobile phones, embedded systems) without aggressive pruning, quantization, or model distillation.

The top-performing Transformer-based models (e.g., Vision Transformers, or ViTs, and a variety of TimeSformer flavors), although also the most computationally intensive in terms of required batch-sized GPU computations and the relative lag among all methods considered in practice, in resource-limited settings.

Acceleration: Autoencoder model-based anomaly detectors are both lighter and there are trade-offs between accuracy and robustness, especially in high-quality and more robust deepfakes.

##### Mobile and edge deployment

For the edge computing scenario (e.g., cameras, mobiles), real-time detection systems should use lightweight architectures. Some promising solutions include:

We prefer variants of the MobileNet and EfficientNet for spatial analysis.
Model quantization, weight pruning and knowledge distillation for minimizing the time and energy involved in the inference.
ROI-based detection mechanism to restrict processing only to facial areas, thus minimizing the computational burden.

However, such custom optimizations may constrain the model’s capacity to see subtle (inferred) artifacts of high-quality manipulations.

##### Scalability in high-volume platforms

Social platforms (such as YouTube, Facebook) need to handle millions of video every day, scalability is an issue. Even in the case of server-side compute clusters, expensive models cannot be run on all videos. Instead, systems tend to employ multistage detection:

Simple or lightweight models are at the first stage, motes up with filters (face detections fast + trivial texture anomaly filters).
Fine-grained models are not applied except on content that has been flagged or on high-relevance types (e.g., political or verified accounts).
Asynchronous processing queues low priority video before determining what is suspicious and what isn't which causes poor real time assurance.

This approach enables efficient but delayed detection, trading off computational cost for coverage.

### Multimedia forensics: applications and challenges

Multimedia forensics is the scientific field dealing with the detection, localization, and analysis of tampered or manipulated digital multimedia content [7]. This area has gained significant attention due to the proliferation of digital media and the increasing sophistication of tools used to manipulate images, videos, and audio. As multimedia content becomes more readily available and easily manipulated, ensuring the authenticity of digital evidence is paramount. The field of multimedia forensics is also used not to simply detect manipulation, but for its importance to provide proof to different fields concerning digital content as is the case of journalism and legal enforcement social media, etc.

Deep learning and artificial intelligence have led to a new era of revolution in multimedia forensics. Such technologies enable much more effective and accurate ways of detecting tampered multimedia content, while promising breakthroughs are surely left behind for researchers and practitioners to explore.

#### Role of deep learning in multimedia forensics

One of the strongest tools in current multimedia forensics is deep learning, especially as it may learn the complex characteristics and patterns hidden within data that exists in the huge dataset, making it easy to perform and tackle different issues relating to tampering with, detecting, localization, and even verification of multimedia authenticity.

##### Tampering detection

In fact, tampering detection is the primary application of deep learning in the area of multimedia forensics. Deep learning models, especially CNNs, find it very easy to differentiate between authentic and tampered media. These models are capable of learning subtle patterns and inconsistencies caused by various ways of tampering such as image splicing, copy-move attacks, or even generation through deepfakes.

For instance, spliced images can be detected through deep learning-based methods, in which a part of an image is replaced by content from another image. These CNNs learn anomalies such as inconsistent lighting, mismatched colors, and unnatural edges in a tampered image through training datasets that comprise images with tampering and without it. Such models can find regions of tampering in an image by learning inherent features of authentic images, which, most of the time, prove to be quite accurate.

In addition, state-of-the-art deep learning approaches such as GANs are used to detect the tampering content generated from the generative model [20]. The ability of GANs to produce highly realistic images or videos enables deep learning algorithms to distinguish between authentic and GAN-generated content, hence increasing tampering detection [24].

##### Localization of tampering

In addition to tampering detection, deep learning techniques are also applied to tampering localization. Localization is pinpointing the region of an image or video that has been altered. This is a significant aspect of multimedia forensics because it provides forensic investigators with more information about the nature of the tampering.

One strategy is applying CNNs for prediction of the likeliness of manipulation in various regions of a multimedia piece. A successful model will tell not just whether tampering has taken place but also which regions of the content are tampered. For instance, in a deepfake video, the face of a person might have been replaced or altered; deep learning models can be used to locate and highlight such changes, which could be very important evidence in legal and journalistic investigations.

Additionally, localization techniques can be used for the detection of copy-move attacks. In this type of attack, a part of an image is copied and pasted elsewhere. Such a model can easily search for the patterns repeated in an image and can mark the parts where the images have been forged.

##### Authenticity verification

Authenticity verification is another very critical area where deep learning comes into the picture [22]. The task is to check if the multimedia content is authentic and has not been tampered with or altered in any form or if it has been tampered with in some form. Deep learning models can be trained on the characteristics of authentic media, and these can then be used to verify unknown content.

For instance, it can be checked using deep learning models whether multimedia content has the consistency of the audio and video signals. On video, this is done both visually and aurally to ensure there are no inconsistencies that would raise suspicion for tampering. Audio anomalies might be unnatural speech patterns, background noise inconsistencies, or other such artifacts.

Deepfake detection [14] forms a very relevant focus area within authenticity verification due to the nearly indistinguishable nature of these media from footage. In consistency detection, this can often go hand-in-hand with RNNs and LSTMs useful in determining inconsistencies and unnatural behavior in the action of facial expression and lip-syncing in relatively small portions where it usually has difficulty catching with the naked human eye. This amounts to a vast quantity of real and fake video data in these models, which train them to identify deepfakes with excellent accuracy.

#### Datasets and benchmarks in multimedia forensics

Most of the power of deep learning models in multimedia forensics relies heavily on the datasets [66] developed for training and evaluation. Several datasets and benchmarks have been designed during the last few years to establish research in this area. It provides a source for large collections of labeled multimedia contents that are trained and tested in the context of deep learning-based models on detection, localization, and verification tasks.

##### Databases for image forensics datasets

Developed are the several image forensic datasets to enhance tampering detection and localization, such as: CASIA, which is possibly the most familiar dataset. Such a dataset features images that underwent tampering. The techniques behind the tampered images include but not limited to the splicing method, copy-move, and also rescaling techniques. This could help researchers with the evaluation and testing of well their models were able to track and locate those tampered image regions [52].

Importantly, this dataset is for the Columbia Image Splicing Detection and has images to detect the spliced variety of the forged images by their type from varied source materials. So far, using deep learning in the research models involves implementing them towards identification and detection along with further localizing how exactly splices might be forged.

##### Video forensics datasets

Video forensics datasets are crucial for training and testing deep learning models that are used in the detection and localization of tampered video content. One of the most popular datasets for deepfake detection is the FaceForensics++ dataset, which contains videos manipulated using state-of-the-art deepfake techniques. The dataset is used to train the models by distinguishing between authentic and deepfake videos, specifically by their capacity to detect minute artifacts introduced by the process of deepfake generation [40].

Another large dataset is the Kaggle DeepFake Detection Challenge Dataset. This dataset holds thousands of deepfake videos that are trained through a wide variety of real and fake videos with deep learning models for deepfake detection. The dataset has proven critical in propelling the advancement of the state-of-the-art in deepfake detection.

##### Audio forensics datasets

Besides image and video forensics, another popular area where the application of deep learning has received significant attention is audio forensics .Perhaps the most well-known resource on hand for spoofed speech detection would be the ASVspoof dataset. The set comprises real samples and synthetic ones and is used as a training scheme for models on spoofed voice and audio manipulation detection [40].

Another one is the VoxCeleb dataset, which has been quite popular for tasks such as speaker verification. Audio recordings of famous persons and public figures are included within this dataset to train models aimed at verifying authentic audio content, besides detecting voice tampering.

#### Multimedia forensics challenges

There are challenges even in multimedia forensics [7] using deep learning that researchers and practitioners need to solve.

##### Adversarial attacks and evasion

The main challenge is the development of adversarial attacks that evade tampering detection models. The attackers produce minor, virtually imperceptible alterations to content and deceive the deep learning-based classifiers into the classification of a tampered message as authentic. These are severe threats because they weaken the integrity of forensic methods in real scenarios.

##### Availability and quality of data

Another is the availability and quality of datasets. There are few datasets for image, video, and audio forensics that could be used; most of the available datasets cover limited scope, thus not fully addressing all types of manipulation methods. Moreover, datasets are not as representative of practical applications, causing models to excel in benchmarking but fail when put into actual practice.

##### Scalability and efficiency

Deep learning models are computationally expensive, requiring lots of processing power and memory. This makes it challenging to deploy forensic tools in real-time applications where speed and efficiency are critical. Scalability is still a significant challenge, especially when dealing with large volumes of multimedia content that need to be processed quickly.

**Fig. 5:**
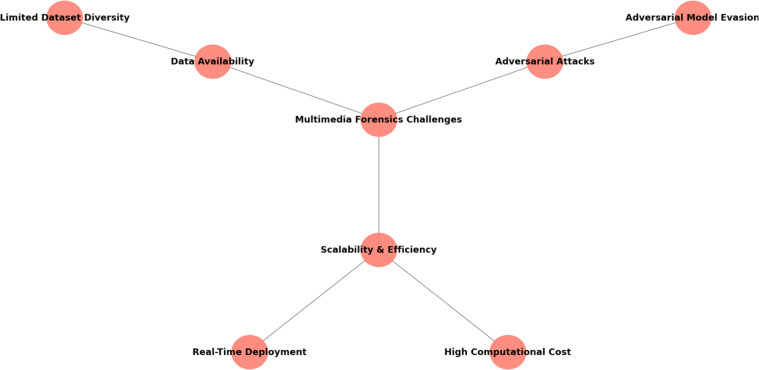
Multimedia Forensics Challenges.

Multimedia forensics is a fast-evolving field that has greatly benefited from the advances in deep learning. Deep learning models have significantly improved the ability to detect, localize, and verify tampered multimedia content with impressive results in tasks such as deepfake detection, splicing identification, and authenticity verification. However, challenges such as the need for much more diverse and high-quality datasets, vulnerability to adversarial attacks, and especially the high computational costs of deploying deep learning models in real-time applications are still open challenges to the field. Greater studies and inventions should be conducted for reliability and effective multimedia forensics against advanced manipulation techniques. Fig. 5 displays The Multimedia Forensics Challenges in Real-World Data.

### Effects on cyber and biometric system

Biometrics has been widely used in modern times as a method to secure applications ranging from mobile device authentications to control borders and secure banking services. Deepfake technology has brought significant focus on issues related to severe security and reliability concerns in the use of biometric systems with attackers exploiting identified vulnerabilities in order to bypass different authentication processes of the system. This article, therefore, gives some potential risks related to deepfakes-based spoofing attacks [70] related to biometrics systems and related case studies around impact on the aspect of cybersecurity [71].

#### Exploiting biometric vulnerabilities through deepfake-based spoofing

The uniqueness of biometric systems lies in physical or behavioral characteristics, such as fingerprints, facial features, retina patterns, voice, or even behavioral traits such as typing speed. They are usually considered more secure than the traditional passwords or PINs because it is very hard to replicate biometrics. However, deepfakes, which have a possibility to create hyper-realistic synthetic media, have come to pose emerging problems to the security of these systems.

In order to fool a biometric authentication system into allowing unauthorized access, deepfake-based spoofing [30] uses machine learning algorithms to produce realistic-looking fake biometric data. Deepfake software, for example, can produce incredibly lifelike pictures, videos, or audio recordings that closely resemble a person's voice or face. These can then be used to bypass facial recognition or voice recognition systems. The reliability of biometric security is seriously threatened by new technologies, it must be said, particularly since these systems are used for serious purposes like financial transactions in critical infrastructure facilities or access to high-security data.

##### Facial recognition systems and deepfake spoofing

One of the most widely used biometric techniques nowadays is facial recognition technology, which is utilized by airports for border control as well as in gadgets like security cameras and smartphone cameras. These methods of identification are practical and efficient, but they are not infallible.

Deepfakes have facilitated attackers to be able to develop hyper-realistic fake facial images or videos that they can use against facial recognition systems [50].

Deepfakes-based spoofing can be conducted in a few ways [60]. One of the techniques is to utilize publicly available pictures or videos of a target and then applying deepfake algorithms to create new images or videos in which the target appears to be performing certain actions, like looking directly into the camera. By using such fake images or videos in conjunction with sophisticated attack tools, an attacker can easily bypass facial recognition systems, gaining unauthorized access to secure facilities, devices, or applications. In addition, because facial recognition systems rely on high-quality images, deepfake attacks can be especially effective in environments where cameras may capture only limited angles or low-resolution images.

##### Voice recognition systems and deepfake spoofing

Voice recognition systems, which are increasingly being used for authentication in mobile devices, banking applications, and virtual assistants, are equally vulnerable to deepfake-based spoofing. Deepfake voice synthesis technology has evolved to the extent that attackers can now create synthetic audio that sounds nearly identical to a person's voice [50]. Such voice replicas can potentially fool even the most advanced automated voice authentication systems.

Voice spoofing is the most significant threat from exploitation through deep fakes, not because of accessing a device physically or system to steal sensitive information or money without authorization. Indeed, an attacker might use some remote deep-fake voice samples to impersonate someone to log into sensitive accounts or financial facilities. This makes the threat worse because it is easy to use and distribute deepfake voice technology. It is possible to create a convincing deepfake voice using just a short sample of a person’s speech, allowing attackers to impersonate individuals without needing lengthy audio recordings or specialized knowledge.

##### Fingerprint and retina scanning systems

Deepfakes are associated majorly with manipulations in the image and audio, but such spoofing would be applicable on other biometrics like fingerprinting or retina scan [58]. Even though deepfake algorithms may not directly replicate the intricate features of fingerprints or retinal patterns, recent advancements—particularly in 3D printing and AI-generated synthetic materials—have enabled the creation of fake biometric data capable of deceiving fingerprint and retina-scanning systems [10].For instance, an attacker might use a 3D printer to create a fingerprint replica of the target by extracting information from publicly available sources or data breaches [58]. Similarly, advanced deepfake technology may be applied in creating synthetic retina scans that replicate the target's unique eye patterns. Although these attacks are more complex and resource-intensive to the ones against facial or voice recognition, it still poses a huge threat especially if an attacker is successful in achieving physical access to the biometric data or can obtain images in high detail about a person's fingerprints or eyes.

#### Case studies of real world attacks and their cybersecurity consequences

The following case studies give an understanding to the world concerning the reality-based impact on the cybersecurity space based on how attackers have taken advantage of the same vulnerabilities presented here.

##### Deepfake voice attack against a CEO in 2019

One of the most discussed cases of deepfake exploitation was in 2019, when cybercriminals used deepfake voice technology to impersonate a CEO in a fraudulent wire transfer scheme. The attackers targeted a UK-based company and used deepfake technology to simulate the CEO's voice, convincing the company's CFO to transfer $243,000 to a bank account under the attackers' control [28].

In this case, the attackers had access to a few audio clips of the CEO's voice, which they used to create a synthetic voice that sounded highly realistic.

Convinced by the voice call's legitimacy, the CFO approved the transfer. The case illustrates the vulnerability of voice verifications and how deepfake technology affects them in high-level business settings, resulting in millions of dollars in losses as well as damage to one's reputation.

##### An american politician in a deepfake video attack

In the second instance, a deepfake video was produced under the pretense of being an American politician in an attempt to sway public opinion. The aforementioned video illustrates how deepfakes are creating ever-growing concerns about misinformation and disinformation, despite the fact that it makes no explicit reference to any hacking or financial scam incidents [6]. Deepfakes can quickly produce believable false reports that sway public opinion, upend established political systems, and spark social unrest.

The attack form has implications for cybersecurity attacks that go beyond financial theft. Deepfakes are used to produce audio or video evidence that contains sensitive information or damages someone's reputation. The argument that attackers can more easily create biometric data to create such contents suggests that digital security systems need to be strengthened [2].

##### Airports at risk of facial recognition hacking

Some airports around the world have adopted facial recognition technology in an attempt to improve the efficiency and speed of passenger security checks. But researchers have shown that deepfakes can fool systems. In one test, for example, a scientist was able to trick facial recognition systems by using deepfake images that closely resembled the face of the person whose face they were mimicking [25]. As a result, someone who is not authorized enters a restricted area of the airport or avoids identity checks.

Such a case study highlights multi-layered authentication mechanisms where feasible to reduce the risks of deepfake-based attacks and poses crucial questions about the effectiveness of security systems based on biometrics as the principle for security in environments.

Deepfake technology presents significant cybersecurity challenges, particularly for biometric systems that rely on voice and facial recognition in addition to other biometric authentication identifiers. Attackers can take advantage of this vulnerability by producing incredibly lifelike synthetic media that can mimic biometric information and grant unauthorized access to private data or locations. The case studies presented illustrate the implications for cybersecurity, which include financial fraud, reputational harm, and even political instability. Organizations must therefore make investments in much more robust security measures, like multi-factor authentication, continuous biometric system monitoring, and the development of deepfake detection software.

To protect their biometric and personal data, people and businesses must be aware of the growing threat posed by deepfake technology and take preventative measures. The future trend of the biometric system and deep fake technology is emphasizing additional research, policy-making, regulation, and further cooperation among cyber specialists and other technologists engaged in the emerging threat.

### Policy recommendations and future trends

Strict policy measures on deepfake's development must be followed in tandem with its actual deployment, as it continues to grow in popularity, technological sophistication, and prevalence [43]. Even though deepfake technology has other admirable uses, such as in academic and research institutions and the film industry, there are also instances of false information, fraud, and data breaches into otherwise secure computer and other systems that demand immediate attention. More significantly, the constantly evolving field of artificial intelligence (AI) offers a plethora of opportunities as well as, more significantly, challenges and solutions for dealing with deepfake problems. This section talks about proposed policy guidelines on regulation of deepfake technology and the new trends in AI research, such as the development of robust detectors and solutions that can deal with ethical concerns.

#### Proposed guidelines for regulating deepfake technology

According to this observation, the conventional system is under tremendous pressure to address the serious risks posed by synthetic media. As a result, governments, regulatory agencies, and tech companies must work together to create a comprehensive set of regulations that will prevent the exploitation of deepfakes while safeguarding their full potential as a potent resource. The article's remaining portion addresses a few important policy recommendations meant to channel this control into practical action in order to properly address the deepfakes technology [72].

##### Creation of insightful legal frameworks and definitions

The creation of precise legal definitions for deepfakes and related technologies will be one of the first steps toward proper regulation. Legal frameworks must distinguish between synthetic media that are used for benign purposes and those that have problems because of improper use. In order to address the issue that deepfakes have brought up, regulatory guidelines will pinpoint areas where lawmakers can step in without limiting innovation [73].

Deepfakes used for entertainment purposes, such as in a movie or video game, would be classified differently than those used for cybercrime or political manipulation. These classifications would help law enforcement bring those who use deepfakes to justice for engaging in harmful or fraudulent activities, such as disseminating misleading information or impersonating someone.

##### Disclosure and transparency requirements

Mandatory disclosure laws must be followed by social media, political, and media deepfakes. Media content that is produced or modified using deepfake technology must be identified as such by its creators. The public would be better able to discern between real and fake content, and greater transparency would be guaranteed. Deepfake detection and flagging systems need to be developed for websites that host user-generated content, like social media networks.

To make it easier to hold individuals and organizations responsible for malicious activities, disclosure of the use of deepfakes needs to be legally enshrined.

Additionally, since the developers won't have to worry about damaging their reputation, fewer deepfakes will be used for unethical purposes.

##### Enactment of strong data protection regulations

As deepfakes extensively draw upon datasets while creating real synthetic media, privacy protection and securing confidentiality regarding private data become considerably unavoidable [72]. Such governments must make serious data protection laws to govern the process of collection and storage of this biometric, voice record data, and manipulated images in developing deepfakes.Strict rules on consent and ownership of data must ensure that the owner of the personal data has control over it especially in applications involving biometric and facial recognition.

Data protection laws must extend to cover synthetic media. For example, legislation can be enacted that criminalizes the unapproved use of personal data in the making of deepfakes, particularly if such deepfakes are used in defamation and fraud [73].

##### International cooperation on deepfake regulation

The deepfakes technology can be developed and shared across borders. Therefore, the regulation of this technology needs to be addressed through collaboration among governments, international organizations, and industry bodies to establish a global standard on responsible use of deepfakes. International agreements, therefore, need to focus on regulating the creation, distribution, and use of synthetic media besides providing penalties for violators of these regulations.

Countries should cooperate on the issue of deepfakes on the global level to address the concerns of cybersecurity threats and the spreading of misinformation. Best practices and technical solutions on the detection and combating of malicious deepfakes shall also be shared.

##### Regulation of deepfake applications in critical sectors

Some industries like finance and healthcare, law enforcement, and national security need to have some supplement controls in order to fight the abuse of deepfakes.

There must be stringent laws and oversight procedures in these sectors where deepfake technology causes financial loss, identity theft, or even threats to national security.

This includes, for instance, enforcing specific policies to adopt advanced authentication in the process of verifying identification within agencies, as well as further bolstering the security of voice and facial recognition systems to prevent spoofing based on deepfakes to satisfy financial needs of institutions.

##### Global policy initiatives and ethical AI frameworks integration

Some global initiatives have offered a platform for ethically based AI systems as part of the development and use of ethical deepfakes. Apart from encouraging responsible innovation, the guidelines [92] have also given builders, regulators, and industry players practical advice on how to stop misuse, particularly in dangerous applications like face impersonation and deepfake development.

###### IEEE's ethically aligned design initiative

IEEE's Ethically Aligned Design Initiative has guiding principles for autonomous and intelligent systems. It calls for transparency, accountability, and human-oriented values in AI, particularly in uses involving high social or psychological impact such as surveillance and disinformation. Such principles emphasize the need for explainability, traceability, and safeguarding human rights when designing and deploying AI systems (IEEE, 2019)[88].

###### European union artificial intelligence act (EU AI act)

The forthcoming (2024) European Union Artificial Intelligence Act (EU AI Act) creates a risk-based categorization of AI systems. Applications of deepfakes are categorized into the "high-risk" or "unacceptable risk" categories depending on the context of use. Such systems should be pre-tested, documented, under human oversight, and be subject to transparency measures (e.g., labeling synthetic content) under the Act. The Act can be used as an international standard for responsible AI regulation (European Commission, 2021; Veale & Zuiderveen Borgesius, 2021 [89], [90].

###### Partnership on AI – responsible practices for synthetic media (2023)

The Partnership on AI (PAI), a multi-stakeholder non-profit organization comprised of academia, civil society, and major tech companies, such as Google, Meta, and Microsoft, issued the "Responsible Practices for Synthetic Media" report in 2023 [91]. The framework offers operational advice for creation and sharing of synthetic media and contains:

(i) Provenance Tagging
  Provenance tagging involves inserting metadata or digital signatures [93] into AI-created (synthetic) content to mark where and when it was created, by whom, and how.
  (a). Purpose:
    - Helps track the source and origin of content.
    - Enables the identification and verification of deepfake or tampered media.
    - Useful in legal, journalistic, and forensic purposes for authenticity verification.
  (b). Example:
    An image created by AI contains a cryptographic hash or embedded metadata that states:
    "Made with Stable Diffusion v2 on 2025–05–01 by User123".
(ii) Disclosure Labeling (such as Visual Watermarks)
  It also involves the inclusion of conspicuous, legible labels on counterfeit media to show that it has been generated or modified with AI.
  (a). Purpose:
    - Prevents the misuse of deepfakes or synthetic media.
    - Facilitates viewers to easily find manipulated media.
    - Promotes openness and trust.
  (b). Common Forms:
    - Visual Watermarks (e.g., "AI Generated" stamped on a video).
    - Audio Disclaimers (e.g., "This voice has been synthesized by AI").
    - Social media post captions or metadata. i.e. A politic parody video contains a watermark in the corner, "This content is AI-generated.".
(iii) Platform Accountability
  Refers to the duty of online platforms (e.g., Twitter, Instagram, YouTube) to moderate, discover, label, and delete toxic artificial media.
  (a). Purpose:
    - Implementing AI-powered detection of deepfakes.
    - Tagging user-uploaded material that is AI-generated.
    - Removing harmful or malicious deepfakes (e.g., disinformation, impersonation).
    - Providing reporting and appeal procedures for inappropriately labeled content.
  (b). Example:
    Facebook flags a false video as such and warns:
    This video was found to have AI-generated content and is misleading.
(iv) Media Literacy Initiatives
  Educational program for instructing the public how to critically examine digital content, especially synthetic or AI-generated content.
  (a). Objectives
    - Inform users on how deepfakes work.
    - Make others doubt authenticity prior to believing and sharing.
    - Enable journalists, teachers, and citizens with the ability to detect manipulation.
  (b). Example Initiatives:
    Government-sponsored or NGO-conducted campaigns

**School Curriculum Additions and Online Fact-Checking Tools**

Online fact-checking tools and media literacy tutorials are becoming essential components of modern education. For instance, a university workshop titled “Detecting Deepfakes: Media Literacy in the Age of AI” teaches students how to identify facial anomalies and metadata indicators in suspicious videos.

The Partnership on AI (PAI) also supports the development of Media Provenance Infrastructure, which uses techniques such as cryptographic hashes and watermarking to trace the origin of digital content and discourage malicious manipulation (Partnership on AI, 2023).

By incorporating these global ethical standards into detection technologies, research policies, and legislative discussions, stakeholders can ensure that synthetic media technologies are brought in line with human values, the law, and democratic accountability. This not only safeguards public trust but also allows for robust-to-abuse innovation in critical areas such as elections, biometric access, and digital evidence.

##### Challenges in enforcing disclosure laws for deepfakes

Enforcing Disclosure Laws for Deepfakes is hard. Passing laws to force deepfake content to be labeled might seem like a good way to regulate against misinformation, but implementing such laws across the globe is very hard to do. Nations have unique law, polity, and culture, and variegated attitudes toward free speech. Criminal or perilous in a certain society, e.g., is legal or promoting in another. A computer-generated deepfake video created in a foreign nation goes viral on the world wide web and is viewed by persons in many foreign nations, raising as a question whose law should be applied and enforced [74].

The bigger issue is jurisdiction — or the authority to govern or punish specific actions. Where a video is hosted on a server in a nation that does not insist on the videos being made public, other nations cannot necessarily force that platform or creator to follow their lead. This in turn gives a reason on its own for bad actors to cheat on regulation by choosing the location from which they wish to distribute [75].

There is also fear that requiring people to label deepfakes could unwittingly generate a climate that favors censorship.

Some countries' governments might abuse these laws to silence critics by accusing them of making false videos, whether or not they are. Civil libertarians worry that if these laws are poorly drafted, they could effectively criminalize free speech and be applied in a way that punishes political activists, journalists, or artists [8].

Disclosure laws are a necessary tool in the fight against deepfake technology abuse, but they should be included in a larger international agreement that offers robust protections for free speech.

Disclaimers on Websites These laws may be implemented, but only if governments around the world support the responsible politics of free expression on the internet. The public may actually accept and trust well-defined regulations that are narrowly focused and strictly scoped in order to combat problematic material, including deepfakes, if the courts, including the Supreme Court, fulfill their duties and protect freedom of speech by removing all needless restrictions.

However, if proactive measures are not taken, the synthetic media's unchecked influence will force governments to enact more restrictive laws. Regulation is a need, not a choice, if these problems get out of hand, such as when harmful or fraudulent content is produced and distributed widely. Many of them worry that new laws might restrict their freedom of expression, particularly in areas like art, satire, and entertainment. In turn, if a regulation is seen as restricting free speech, the public frequently opposes it on almost any level out of fear of censorship.

#### Upcoming developments in AI research to strengthen detection and get past ethical barriers

Research on artificial intelligence is rapidly expanding, focusing on deepfake detection systems [101] and bringing ethical concerns about synthetic media into the mix. In order to prevent deepfake misuse, the researchers are still searching and experimenting with various approaches, but they make sure that ethics are at the forefront of most mainstream AI development. The following are a few of the most exciting new directions in AI research:

##### Detection algorithms and AI-Based solutions for deepfake

The development of AI-driven detection algorithms is arguably the most researched topic in the fight against deepfakes [12]. The development of machine learning systems that can analyze media content and identify minute discrepancies or clues left by deepfake technology is underway. The purpose of these systems is to identify the abnormalities in pixel patterns, lighting, facial expressions, and audio signals that are characteristic of deepfake media.

Using CNN and RNN models, deepfake detection algorithms [100] have been rapidly becoming more complex. These algorithms will analyze every type of media, whether it be audio or video, in great detail. Furthermore, some employ technologies such as adversarial networks, which expose the AI system to both authentic and fraudulent content in order to train it to recognize deepfakes. If these AI-powered detection systems are integrated into online social media, news websites, and video-sharing platforms, they have a lot of potential to stop the spread of malicious deepfakes. These websites' real-time deepfake detection allows them to automatically flag dubious content and halt it in its tracks.

##### Ethical AI and responsible development

With the improvement in the capabilities of AI and deepfake technology, ethics in AI research have become a major focus area. For instance, Pawelec [8] explores how professional deepfake developers perceive ethical boundaries and governance potential in their work.. AI-based deepfake detection systems need to be transparent, accountable, and in accordance with the values of society. Therefore, there are many researchers engaged in the task of creating ethics and guidelines in the development of AI with special importance given to privacy, fairness, and the responsible use of synthetic media.

Design should respect people's privacy and avoid infringing upon their rights; for instance, AI-based tools for detecting deepfakes have to be very calibrated to ensure not to indirectly discriminate against any individual or group of people. Furthermore, research in the direction is also done regarding ensuring that freedom of speech of people or proper creative usage does not get infringed upon with regard to using such technology for detection by AI systems.

##### Collaboration between industry and academia

One more trend, which is emerging in AI research, relates to deepfakes.

In this regard, it seems more important than ever for industry and academia to work together to address the problems posed by deepfakes. Tech companies, academic institutions, and research centers are coming together to work on new detection system technologies, exchange datasets for training models, and evaluate current solutions. Platforms and organizations at risk of deepfakes can adopt more quickly if they work together with the industry.

Politicians, ethicists, and AI scientists must also communicate with one another in order to reach a compromise between innovation and regulation. In order to use AI responsibly and safely, an interdisciplinary approach spanning these and other disciplines will create the ethical safeguards surrounding deepfakes.

Enact laws addressing deepfakes and sophisticated AI detection tools to pave the way for the further reduction or eradication of some of the synthesis media production that appears to be a threat. It will result in clear legal frameworks, a stringent disclosure ruling, and a more stringent data protection law, all of which will contribute to the development of a safer but more responsible environment for responsible innovation. As AI develops further into the future, ethical issues are known to be at the forefront of real advancements in the field. By creating more reliable systems and regulatory frameworks, collaboration between businesses, governments, and academic institutions makes it possible to detect deepfakes even more effectively and robustly.

Regulation of deepfakes and advancements in AI research will ultimately be able to shield society from these harmful effects of advanced technology.

## Conclusion

Even though deepfakes technology has a lot of exciting potential, there are some very real risks associated with it. As such, we must work together to address these issues. The latest developments in deepfake creation, detection techniques, multimedia forensics, and related cybersecurity risks have all been examined in this comprehensive review. The technical advancements and urgent vulnerabilities in the current synthetic media landscape are highlighted in this work by combining recent developments in biometric spoofing defenses, AI-based detection systems, and ethical AI frameworks.

This review does, however, acknowledge some limitations. Since the deepfake ecosystem is changing so quickly, many of the detection models covered here run the risk of becoming outdated as new generative methods (like diffusion models and real-time video synthesis) are developed. Furthermore, the generalizability of detection models across various populations and real-world conditions is limited by the absence of standardized evaluation benchmarks and the inherent biases in existing datasets.

Therefore, the following practical directions should be given priority in future work:

(i) Establishment of Inclusive and Standardized Datasets
  Urge international consortia to create benchmark datasets that reflect a range of environments, modalities, age groups, and ethnicities. These kinds of datasets ought to be publicly available and annotated for realism, quality, and type of manipulation.
(ii) Investment in Multimodal and Cross-Domain Detection Systems
  Future systems must leverage joint analysis of visual, auditory, and textual cues, and be stress-tested across domains (e.g., surveillance, livestreams, compressed social media content) to improve robustness and generalization.
(iii) Regulatory Collaboration at a Global Scale
  Develop and harmonize international regulatory frameworks that mandate synthetic media disclosure, protect biometric privacy, and ensure ethical AI deployment. A global standard will help address misuse across borders and platforms.
(iv) Adversarial Robustness and Real-Time Scalability
  Research must also focus on defending detection systems from adversarial attacks and reducing their computational cost for deployment on mobile and edge devices, especially in high-risk domains like banking, surveillance, and elections.
(v) Transparency and Accountability in AI Detection Systems
  Promote explainability in detection models to support legal admissibility and public trust. Detection frameworks should offer interpretable outputs and maintain audit trails of flagged content.

In conclusion, countering deepfakes is not just a technical challenge but a societal imperative. A combined effort across technology, ethics, policy, and public education is essential to ensure that advances in synthetic media are met with equally innovative and responsible detection mechanisms.

## Ethics statements

This study did not involve human participants or data collected from social media platforms. All reviewed datasets are publicly available and anonymized. No data redistribution policies were violated.

## Supplementary material and/or additional information [OPTIONAL]

None.

## CRediT authorship contribution statement

**Sonam Singh:** Conceptualization, Methodology, Writing – original draft, Writing – review & editing. **Amol Dhumane:** Supervision, Validation, Writing – review & editing.

## Declaration of competing interest

The authors declare that they have no known competing financial interests or personal relationships that could have appeared to influence the work reported in this paper.
